# Post-genomic era in agriculture and veterinary science: successful and proposed application of genetic targeting technologies

**DOI:** 10.3389/fvets.2023.1180621

**Published:** 2023-08-03

**Authors:** Ali Mazloum, Maxim Karagyaur, Roman Chernyshev, Antoinette van Schalkwyk, Ma Jun, Fu Qiang, Alexander Sprygin

**Affiliations:** ^1^Federal Center for Animal Health, Vladimir, Russia; ^2^Institute for Regenerative Medicine, Medical Research and Education Center, Lomonosov Moscow State University, Moscow, Russia; ^3^Agricultural Research Council-Onderstepoort Veterinary Institute, Onderstepoort, South Africa; ^4^Department of Biotechnology, University of the Western Cape, Bellville, South Africa; ^5^School of Life Sciences and Engineering, Foshan University, Foshan, China

**Keywords:** genome editing, CRISPR-Cas, genetically modified organisms, vaccine, virus loxP mouse Cre mouse

## Abstract

Gene editing tools have become an indispensable part of research into the fundamental aspects of cell biology. With a vast body of literature having been generated based on next generation sequencing technologies, keeping track of this ever-growing body of information remains challenging. This necessitates the translation of genomic data into tangible applications. In order to address this objective, the generated Next Generation Sequencing (NGS) data forms the basis for targeted genome editing strategies, employing known enzymes of various cellular machinery, in generating organisms with specifically selected phenotypes. This review focuses primarily on CRISPR/Cas9 technology in the context of its advantages over Zinc finger proteins (ZNF) and Transcription activator-like effector nucleases (TALEN) and meganucleases mutagenesis strategies, for use in agricultural and veterinary applications. This review will describe the application of CRISPR/Cas9 in creating modified organisms with custom-made properties, without the undesired non-targeted effects associated with virus vector vaccines and bioactive molecules produced in bacterial systems. Examples of the successful and unsuccessful applications of this technology to plants, animals and microorganisms are provided, as well as an in-depth look into possible future trends and applications in vaccine development, disease resistance and enhanced phenotypic traits will be discussed.

## 1. Introduction

Targeted genome engineering enables the modification of specific genomic loci at predetermined sites to generate novel plants, animals, and microorganisms by bypassing the procedure of using random mutagenesis and long-term selection (1, 2). Understanding the functional significance of individual coding and regulatory genes creates unprecedented opportunities. These technologies make it possible to revolutionize agriculture and industry to solve research and practical problems in the areas of food and biological safety. The development of these technologies will enable us to further understand the function of individual genes, which can be exploited to improve nutrition and the taste of food, and increase the resistance of agricultural animals and plants to infection and parasites (3–5).

Among the main approaches to directly modify the genome, two technologies can be distinguished: ectopic gene insertion (cis- and trans-genesis) and editing the nucleotide sequence (6, 7). Each of these strategies is radically different from the previous approaches of induced mutagenesis and crossing that were once widely used. Their use required a clear understanding of the function of individual genes as well as their interactions and regulation. Thus, the use of direct genomic modification technologies is practically devoid of the element of randomness, and the resulting organisms, with a high degree of probability, acquire and transmit the desired properties and characteristics to their offspring. Each of these approaches has certain features, advantages and limitations, successes and failures, of which the most relevant will be discussed in this review.

## 2. Genome editing before the advent of CRISPR/Cas9

Genetic engineering was first introduced into scientific practice in the 1970s, even before the advent of widespread modern nuclease-based gene editing platforms; however, a number of technical shortcomings limited its widespread use. The limiting factors of the methods initially used were relatively low efficiencies and specificity (depending on the specific object of research) as well as significant material and time costs.

At the early stages of genetic engineering, genome modification was based upon the natural mechanism of homologous recombination (very low efficiency–up to 0.001%), when a vector (usually a recombinant plasmid) containing a DNA construct corresponding to the sequence of the target gene with the necessary modifications exchanged nucleotide sequences with the intact target gene (8). The revolutionary work of Rouet et al. demonstrated the possibility of introducing double-strand breaks into the chromosomes of mouse cells of the transplanted COS1 cell line using an expression system of the rare-cut nuclease I-Sce I with amazing efficiency, up to 82% (9, 10).

**Figure 1:**
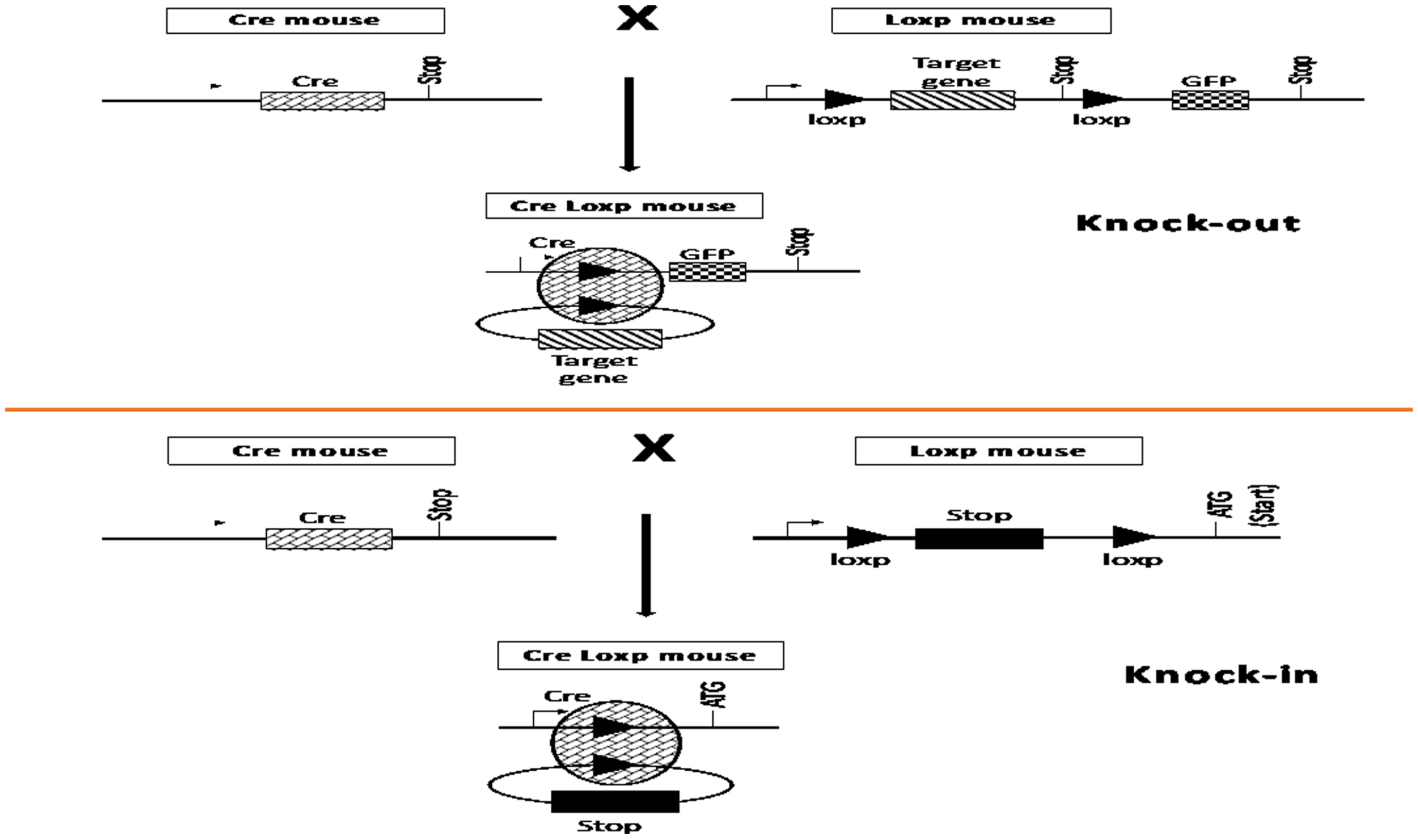
Mechanism of action of site-specific recombinases in mouse cells. This figure is based on previously published work (18).

The discovery of site-specific recombinases (SSR) of bacteriophage P1 and baker’s yeast *Saccharomyces cerevisiae* introduced the method of complete gene deletion into the practice of genome editing (11, 12). The two most common types of SSR are the Cre-loxP and Flp-FRT systems. Cre recombinase is an enzyme first discovered in bacteriophage P1 that excises a DNA fragment by homologous recombination between highly specific flanking sequences, known as Lox-P sites (13, 14). The Flp-FRT system operates in a similar manner; however, the Flp recombinase discovered in baker’s yeast recognizes FRT sequences (15, 16). During hybridization of cells expressing SSR and cells containing the gene of interest flanked by loxP or FRT sites, the target gene may be cleaved and inactivated (Figure 1) (17, 18). The main disadvantage of this method is the need for preliminary integration of loxP and FRT sites within the genome region to be deleted. Therefore, its main application was the creation of genomic modifications for basic animal research (19). The targets in this case, as a rule, were marker genes encoding green fluorescent protein (GFP), β-galactosidase, antibiotic resistance enzymes, and others (20).

**Figure 2:**
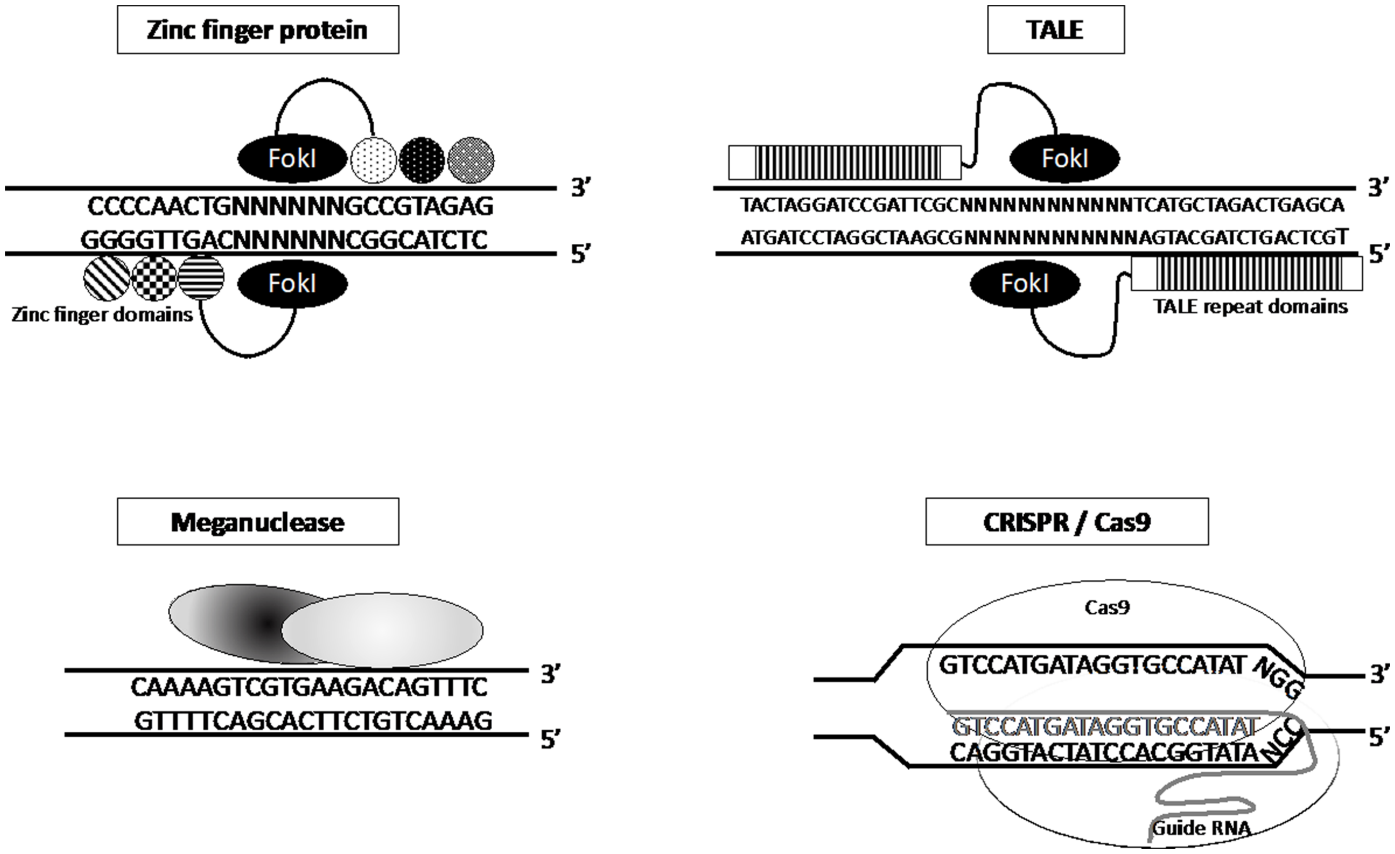
Mechanism of action of different genetically engineered nucleases for DNA restriction (22).

Because it was found that the introduction of a double-stranded DNA break markedly increases the efficiency of genome editing, the search for endonucleases capable of generating such breaks within a desired region of the genome began. As a result, meganucleases were used for these purposes and a number of artificial nucleases were developed including zinc-finger nucleases (ZFNs) and transcription activator-like effector nucleases (TALENs). A feature of these nucleases is the recognition of the target DNA sequence due to amino acid residues, which required time-consuming optimization of the accuracy and efficiency of these systems. In 2012, the CRISPR/Cas9 bacterial “immunity” system was proposed for genome editing, which targets a region of the genome based on Watson-Crick interactions between the target region of the DNA and crRNA/gRNA, which is part of the CRISPR/Cas9 effector complex. The introduction of a double-stranded DNA break triggers the repair process, which includes non-homologous end connection (NHEJ) and homologous directed repair (HDR). NHEJ directly connects the ends of the cleaved DNA, whereas HDR uses a homologous sequence as a matrix to restore missing DNA sequences at the break point (21). A brief demonstration of the mechanism of action of these four nuclease systems is presented in Figure 2.

The first kind of nucleases that were used were Meganucleases, which are a highly specific enzymes characterized by an ability to recognize and cut longer DNA sequences from 14 to 40 base pairs (Figure 2) (22). There are five meganuclease families based on their structures: LAGLIDADG, His-Cys box, HNH, PD-(D/E)XK and GIY-YIG (23). The LAGLIDADG family of proteins exhibits conserved features, of which I-CreI, I-CeuI, I-AniI, I-SceI, I-PfuI, and I-DmoI, are the most widely used (24, 25). Because of their high specificity for the DNA target site, the number of candidate target genes for genome editing is relatively low, which requires the identification of new meganucleases for genetic modification of other genes. Currently, tens of thousands of such enzymes are available; however, identifying specific meganucleases for each purpose (modification) is a significant obstacle when conducting research (26).

The second type are Zinc-finger nucleases (ZFNs), a synthetic endonucleases constructed by fusing the DNA-binding domains of transcription factors with the nuclease domain of FokI restriction enzyme (27). Each DNA-binding domain can recognize 3 nucleotides and when combined into tandem structures of 3–6, the DNA-binding domains of individual ZFNs are able to recognize 9–18 pairs of nucleotides in the target DNA. ZFNs involve the use of a pair of editors designed in such a way that each pair recognizes DNA sites in the forward (5′-3′) and reverse (3′-5′) strands of the target gene flanking the area selected for editing. With this design, the nuclease domains of FokI dimerize and introduce a double-strand break in DNA (Figure 2) (28, 29). Despite a number of advantages, the use of ZFN is limited because of the high cost and complexity of designing and testing the editors, the limited choice of the target site, and the possibility of non-specific DNA cleavage.

Transcription activator-like effector nucleases (TALEN) are the third type of artificial programmable endonucleases, created by fusing transcription activator-like effector (TALE) DNA-binding domains to the DNA cleavage domain of FokI endonuclease (Figure 2). TALEs can be engineered to bind to practically any desired DNA sequence, so when combined with a nuclease, DNA can be cut at specific locations. The TALE DNA-binding domain contains a repeated highly conserved 33–34 amino acid sequence with divergence at the 12th and 13th amino acids (30). These two positions, referred to as the Repeat Variable Di-residue (RVD), are highly variable and show a strong correlation with specific nucleotide recognition (31).

The main advantage of the TALEN design over ZFN is the greater number (>18 bp) of binding nucleotides. To date, there are approximately 20 constructed RVDs, 7 of which are used in 90% of the cases (32). Among other advantages of TALENs is the ability to edit a wider range of DNA sites compared with ZFNs, which are determined by the sequence of the DNA-binding domain. Nevertheless, TALENs have disadvantages manifested both in off-targeted binding effects and the difficulty in delivering enzymes to target cells because of their high molecular weight (33, 34).

The fourth type of nucleases are CRISPR/Cas, which revolutionized the science of genetic modification in the past decade, and will be subsequently discussed in more detail.

## 3. CRISPR/Cas9-mediated genome editing

**Figure 3:**
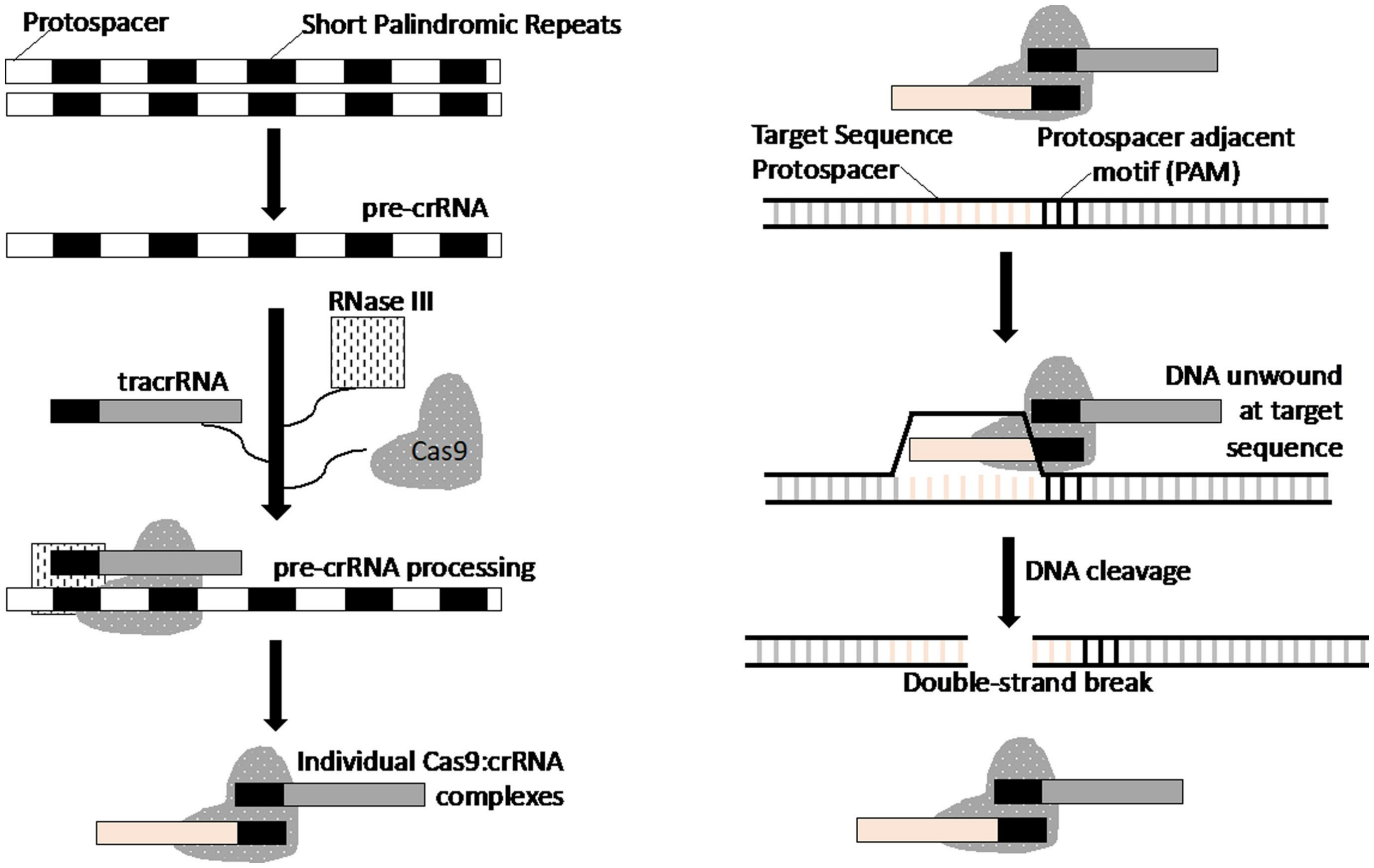
Schematic representation of the mechanism of the CRISPR-Cas system for double-stranded DNA cleavage. This figure is based on previously published work (21).

A recent and revolutionary genome editing tool is the CRISPR/Cas9 bacterial “immunity” system, which is based on the guiding ability of short RNAs and nuclease activity of the CRISPR-associated Cas9 enzyme. When combined, the complex (Cas9/gRNA) can cleave DNA at almost any desired site (Figure 2) (35, 36). In nature, CRISPR (clustered regularly interspaced short palindromic repeats) is an adaptive immunity mechanism for members of the Archaea and bacteria kingdoms. It consists of two key components; CRISPR array and CRISPR-associated nuclease. The CRISPR array stores genetic information pertaining to previous phage or plasmid infections, thus acting as a genetic “memory.” It consists of tandem nucleotide repeats (25–36 bp in length) separated by unique sequences of approximately the same length, also known as spacers. The spacers are acquired from the genome of parasitic elements during prokaryote infection and become the basis of prokaryote adaptive immunity, as they guide the CRISPR-associated nuclease (the second component of CRISPR/Cas9 system) to destroy the parasite genome during re-infection. A short RNA guiding CRISPR/Cas9 system that targets DNA is known as crRNA (CRISPR RNA). The genes encoding Cas proteins are often located in close proximity to the CRISPR template (Figure 3) (21, 37).

Three types (I–III) of CRISPR systems have been identified across a wide range of bacterial and archaeal hosts. Each system comprises a cluster of CRISPR-associated (*Cas*) genes, noncoding RNAs, and a distinctive array of repetitive elements (direct repeats). The Type II CRISPR system is well-characterized, consisting of the nuclease Cas9, the crRNA array that encodes the guide RNAs, and a required auxiliary trans-activating crRNA (tracrRNA) that facilitates the processing of the crRNA array into discrete crRNAs and mediates the crRNA interaction with the Cas9 apoenzyme. The synthetic crRNA analog obtained by fusion of crRNA and trans-crRNA is known as a single guide RNA (sgRNA or gRNA). Because of its convenience and easy customization, CRISPR/Cas9 technology has been used for genome editing of target cells. Since 2010, multiple modifications and improvements to this system have been implemented to make it easier to use and available to any researcher (38). This system enables one to control gene expression, make precise genome modifications, and even trigger chromosomal recombination.

As mentioned previously, CRISPR-Cas technology relies on the interaction between the crRNA/gRNA and target DNA (RNA–DNA interaction), which determines the exact position where Cas nucleases can perform double-strand DNA cleavage. Changing the sequence of the sgRNA changes the specificity of the CRISPR/Cas9 system. Because the synthesis of a new crRNA/gRNA is easy, CRISPR/Cas9 has a number of advantages compared with ZFNs and TALENs, such as the simplicity of design, versatility with respect to any target, and the ability to modify several genomic sites simultaneously (multiplex editing). The only significant disadvantage of CRISPR/Cas9 is the high molecular weight of the Cas9 nuclease, which can impede delivery of the genome editing components into cells. However, various easy-to-use delivery methods (e.g., lentiviral expression system, liposomes, electroporation, transfection by recombinant plasmids) have been developed to overcome this limitation (39, 40). A comparison of the advantages and disadvantages of the four methods for nuclease genome editing is presented in Table 1 (41). Based on the majority of the parameters evaluated in Table 1, CRISPR/Cas9 technology is the superior or most preferred technology.

**Table 1:** Comparison of parameters for various methods of genetic editing using nucleases (41).

Parameters	Method of genome modification using nucleases			
	ZFNs	TALENs	Meganucleases	CRISPR/Cas
Versatility	Low	Medium	Low	High
Cost	Low	High	Low	Low
Cytotoxicity	++	+	-	-
Specificity	Low	Medium	High	High
Effectivity	Medium	Medium	Low	High
Construction	Moderately difficult (requires specific enzyme for each DNA fragment)	Difficult (Requires the synthesis of a highly specific enzyme for DNA and a method for delivering the protein to the target cell)	Difficult (requires it is necessary to select a specific restriction site for each gene)	Moderately easy (Requires synthesis of RNA-guides and delivery of Cas9 to target cell)
Multiplex genome editing	Low probability	Low probability	Impossible	Possible
Preparation of additional helping factors	Not required	Requires delivering the enzymes to the target cell	Not required	Requires synthesis of RNA-guides and delivery of Cas9 to target cell
Probability of NHEJ	Low	Low	Low	Medium
Probability of HR	Low	Low	Low	Medium

## 4. CRISPR-Cas9 applications

CRISPR -Cas9 technology facilitates knockin–knockout strategies to study and determine the influence of a particular gene on the virulence of a virus or its replication as well as the phenotypic traits of animals or prokaryotes. The results of such modifications are usually associated with one of two possible outcomes: knockout - deletion of any gene from the genome, or knockin–introduction of a new gene into the genome of a virus or animal. As a result, the implementation of a DIVA strategy (differentiation of infected from vaccinated animals), in which the gene is inactivated by integrating a marker gene (e.g., encoding a green or red fluorescent protein), facilitates the selection of the recombinant organism.

The major advantages of CRISPR/Cas 9 is the ease and versatility of design and cloning to generate guide RNA (gRNA) libraries for large-scale genetic screening. Genomic screenings enables the discovery of the function of new genes and to determine their role and mechanism of action within living cells. Before CRISPR/Cas9, genomic screenings were performed using two main approaches: “turning on” gene expression using a cDNA library and “turning off” genes by RNA interference (42). The efficacy of these approaches was rather restricted, whereas contemporary gRNA libraries enable one to edit not only the whole genome, but a subset of genes involved in a specific signal or metabolic pathway as well as ncRNAs. Moreover, no Cas9-related cytotoxicity has been reported and its activity depends solely on the complex of NHEJ proteins responsible for DNA repair following double-strand breaks. Currently, CRISPR/Cas 9-based screenings are used to analyze pathological processes, such as carcinogenesis, metastasis, and inflammation (21).

Various scientific groups around the world are conducting research on the development of prevention strategies for various animal diseases (e.g., African swine fever, classical swine fever, Marek’s disease, capripoxvirus infections, Aujeszky’s disease) in an effort to create a safe and effective biological product or vaccine that provides long-term protection against infection.

### 4.1. Application of CRISPR/Cas9 during the production of healthy animals with useful economic traits

The increasing global population depends on the effective and enhanced productivity of livestock and poultry for food. One of the best strategies to increase the efficiency of food production is to select farm animals with the most desirable traits, either through extensive selective breeding campaign or by modifying the genetics of these animals (43). Microinjection of the structures required for CRISPR/Cas9 function, directly into zygotes results in a high probability of obtaining offspring with the desired recombinant traits. The CRISPR/Cas9 construct is capable of inactivating genes and creating site-specific mutations by cytoplasmic injection, pronuclear injection, or electroporation (44).

Because CRISPR/Cas9 has become an important tool for genetic engineering, it has been used to produce changes and correct undesirable defects in specific genes to improve the quality of livestock (e.g., cattle, pigs, horses). A large number of animals with edited genomes have been created. These include a pig with an altered myostatin gene, resulting in larger muscle mass, or a pig with resistance to the reproductive and respiratory syndrome virus, and transgenic cattle resistant to tuberculosis (45–47). Recently, scientists from China has created three different pig lines that are resistant to the classical swine fever virus (48–51). When studying the productivity of genetically modified livestock, significant improvements have been achieved with respect to meat yield, disease resistance, and other desirable qualities (52).

### 4.2. The use of CRISPR/Cas9 in the modification of viral genomes

Virus replication depends on numerous factors in the target host cells. Due to the obligate host-dependent replication of viruses, it is more difficult to apply genome editing tools to the virus genome than compared with the editing of the eukaryote host genome. Success in generating novel viral mutants has been achieved with CRISPR/Cas9 in the absence of viral replication in the cell and, as a consequence, decontamination of cell cultures and sensitive organisms. Recently, various studies have successfully used CRISPR/Cas9 technology to obtain attenuated viral variants (viruses with both DNA- and RNA-containing genomes) as vaccine prototypes (53, 54).

#### 4.2.1. Editing DNA viruses using CRISPR/Cas9

The CRISPR/Cas9 system has been successfully applied to DNA-containing viruses, such as the smallpox vaccine virus (VACV) (55, 56), human polyomavirus 2 (JCPyV) (57), African swine fever virus (ASFV) (58–60), avian adenovirus 4 (FAdV-4) (61), Aujeszky’s disease virus (62, 63), and Marek disease virus (64) (Table 2). CRISPR/Cas9 technology guided by gRNA provides significantly greater efficiency for creating mutant VACVs without obvious side effects in recipient organisms compared to using a site-specific recombinase system. Additionally, it provides a marker-free system that can be used to efficiently construct VACV vectors carrying therapeutic genes in the TK or N1L regions (55, 56). A system was established to make changes to the non-coding region and the open reading frame (ORS) of the “late” gene in the genome of human polyomavirus 2, which subsequently inhibited viral replication and protein expression (57). The genomes of ASFVs were altered between positions 71 and 78 in the phosphoprotein p30 gene, encoded by the CP204L gene. This resulted in the mutated ASFVs not displaying the classical cytopathic effects when cultured in the infected wild boar lung cell line WSL (58). Additionally, the complete deletion of the gene encoding the immunogenic protein A238L (5EL) of the ASF virus genotype IX was the basis for implementing a differentiation between infected and vaccinated animal (DIVA) strategy for ASF (59). The CRISPR/Cas9 system was successfully applied to generate a unique nucleotide deletion (1966DEL) within the avirulent strain KR5 of the avian adenovirus 4, between ORS 42 and ORS 43 confirmed the absence of virulent function (61).

**Table 2:** Genetically modified DNA viruses using CRISPR/Cas9.

Virus	Serotype/Strain	Modified gene	Type of modification	Reference
Aujeszky’s disease virus	HNX	TK, gE	Deletion	(62)
	BarthaK61	EP0, UL50	Insertion	(63)
African swine fever virus	Georgia 2007/1	8-DP	Deletion	(60)
	-	Cp204L	Deletion	(58)
	ASFV-Kenya-IX-1033	A238L (5EL)	Deletion	(59)
Marek disease virus	RB-1B GFP	UL6/UL19/UL27/UL30/ UL49/ICP4/UL25	Deletion	(64)
Human polyomavirus 2 (JCPyV)	-	«late» gene	Deletion	(57)
Avian adenovirus4 (FAdV-4)	KR5	ОРС42/43 (1966DEL)	Deletion	(61)

#### 4.2.2. Editing of RNA-containing viruses using CRISPR/Cas9

Currently, the CRISPR/Cas9 system is primarily used for editing genes of DNA-containing viruses, but technical obstacles related to its use for the mutagenesis of RNA-containing viruses remain. The recently discovered Cas9 nuclease from the Gram-negative bacterium *Francisella novicida* (FnCas9) is capable of targeting endogenous bacterial RNA. This FnCas9 can be directed by an engineered RNA-targeting guide RNA to target and inhibit a human +ssRNA virus, hepatitis C virus, within eukaryotic cells. This work revealed a versatile and portable RNA-targeting system that can effectively function in eukaryotic cells and be programmed as an antiviral defense (65). The recently developed CRISPR/Cas13a system effectively modified the NS3 region of the Dengue virus and the replication of the recombinant virus in mammalian cells (66). The loss of infectious activity of the Porcine reproductive and respiratory syndrome virus (PRRSV) resulted from the inactivation of the viral genes, ORC5 and ORC7, using the CRISPR/Cas13b system; thus, the cleavage of viral RNA was observed in virus-sensitive cells (67). These data demonstrate a new and effective technology for future gene editing of RNA-containing viruses.

### 4.3. The use of CRISPR/Cas9 in the creation of vector vaccines

Orthopoxviruses (OPXV) are suitable viral vectors for gene expression and therapy because they have a large transgenic capacity up to 25,000 base pairs (kbp) (68), a wide range of hosts (including humans) (69, 70), and they are capable of stimulating both long-term cellular and humoral immune responses against the viral vector (−s) antigen (s), despite the existing vector immunity (68, 71). They are also thermostable, easy to store, transport, and use, which ultimately ensures their high profitability in commercial production systems. The first OPXV successfully used as a vector was the smallpox vaccine virus (VACV), which was used as a vaccine to fight smallpox in humans. Because of the successful use of recombinant VACV-encoding hepatitis B antigens to stimulate the immune system against hepatitis B (72), replication-deficient orthopoxvirus-modified vaccinia virus Ankara (MVA) and NYVAC was adopted for constructing recombinant vectors and vaccines against various human and animal diseases (73, 74).

Recombinant OPXV can encode antigens for one or more infectious agents, including antigens and genes encoding specific immune-stimulating factors, such as cytokines or chemokines (75). Recombinant OPXV can be constructed by homologous recombination between transfected heterologous DNA and replicating viral DNA (76), *in vitro* ligation (77), or recombination of artificial bacterial chromosomes (78). Commonly used insertion sites include the thymidine kinase (TK) gene, the hemagglutinin (HA) gene, the intergenic region between the F12L and F13L genes, and sites of natural deletions in the OPXV genome (especially with respect to MVA) (76).

As a rule, these systems require multistage operations with low efficiency. The creation of candidate recombinant vaccines by homologous recombination is limited due to low recombination efficiency (<3%), the complexity of processes (for example, the creation of a plasmid with a transgene and selection of a recombinant virus), the instability of the transgene, the need for 200–500 bp flanking DNA sequence (which promotes recombination to non-target sites), and the absence of simultaneous multiple editing of several genes (76, 77, 79–82). Using CRISPR/Cas9, scientists have been able to circumvent all of these limitations to accelerate the process of obtaining recombinant viruses as vectors during the transfer of foreign genes in the prevention of various animal diseases.

To date, several recombinant vaccines and vectors based on OPXV have reached different stages of clinical trials. Various vaccines and vectors are based on VACV, MVA, NYVAC, raccoon poxvirus, lumpy skin disease virus (LSDV) and modified strains of OPXV Tian Tian smallpox vaccine (MVTT). They target malignant neoplasms (e.g., prostate, skin, colorectal cancer, breast and ovarian cancer) (83–88) and infectious diseases (e.g., AIDS, malaria, Ebola, tuberculosis, hepatitis, flu). In many parts of Europe, Canada, and the USA, the recombinant vaccine VACV–Raboral V-RG is widely used for the eradication of rabies virus in wild fox populations (76, 89). Several vector vaccines based on OPXV have been used to prevent various animal diseases (Table 3) (88).

**Table 3:** Current vaccines based on orthopoxviruses (88).

Orthopoxvirus strain	Recombinant vector	Host	Disease	Integrated gene
Vaccinia	PROSTVACV/ TRICOM; Vaccinia-B7.1	Human	Prostate; Melanoma cancer	Prostate specific antigen; and co-stimulatory T-cell molecules
	PANVAC-V	Human	Colorectal, thoracic and ovarian cancer	Transgenes for the tumor-associated antigens epithelial mucin 1 and carcinoembryonic antigen
	Raboral V-RG	Wild animals (red fox, coyote, raccoon dog, cats)	Rabies	Rabies virus glycoprotein G
NYVAC	NYVAC- CDV-H/F	Ferrets	Plague of dogs	Canine distemper virus haemagglutinin (H) and fusion (F) protein genes
	NYVAC-EHV-1-vP1014	Equine	Equine rhinopneumonia	Immediate early gene (gene 64) of equine herpesvirus-1 (EHV-1)
	NYVAC-PRV- gII/gp50	Swine	Pseudo-rabies	Pseudorabies virus glycoproteins *gII* and *gp50*
Racoon poxvirus	RCN /rabies-G	Cats	Rabies	Rabies glycoprotein G

### 4.4. The use of CRISPR/Cas9 for the study of the interaction viruses with host cells

Recently, a genome-wide screen using CRISPR/Cas9 was conducted to identify host factors that may potentially participate in the replication of certain viruses, such as SARS-CoV-2 (90), HIV (91), arthritic alphaviruses (92), Coxsackie virus (93), Venezuelan equine encephalitis virus (VEEV) (94) and influenza virus (95). For the influenza virus, it was demonstrated that inactivation of specific host genes, such as DOCK5, Annexin-A1, IFIT2, IRF7 and ZDHHC22, can induce protection against cell death during infection (96–100). Because of the high throughput and precise editing features of the CRISPR/Cas9 screening system, one can quickly and accurately identify specific genes and proteins involved in the pathogenesis of viruses. The relative ease of use and reproducibility of the CRISPR/Cas9 system makes it a powerful tool for studying virus-host interactions and identifying new antiviral targets.

### 4.5. The use of CRISPR/Cas9 to create bacterial producers of recombinant proteins

CRISPR-Cas9/Cas12a are well-studied nucleases optimized for the production of recombinant bacteria, which benefits the production of food, vitamins, and medicines. Bacteria may be used as biological “factories,” which can readily manufacture products using cheap raw materials.

A list of genetically modified bacteria using CRISPR/Cas technology are listed in Supplementary Table S1.

## 5. Comparison of prospects for the development and application of genetic targeting technologies in the future

As described earlier, cis- and trans-genesis technologies enable the introduction of new genes into the genome to impart fundamentally new qualities on the host cell; for example, pest resistance, cold, or drought tolerance (101–103). Such foreign genes can also be introduced into the recipient genome using genome editing technologies (104). Genomic editing, for the most part, has a less radical effect on the physiology of the modified organism, because the changes are usually precise and minimal.

The genome and transcriptome of each organism may be considered a balanced system over the course of evolution where a significant change in the nucleotide sequence (more pronounced than spontaneous mutagenesis) could change the phenotype, or introduce foreign genes that may cause an imbalance to this system. Interference between existing and newly acquired molecules, signaling, and metabolic cascades can be unpredictable, which can reduce the value of productive Genetically Modified Organisms (GMOs) or render them potentially dangerous to the environment or for food consumption (105, 106).

A striking example of the unpredictable consequences of the genomic modification of a productive organism was the creation of GMO soybeans resistant to the herbicide glyphosate (Monsanto) (107). The resulting GMOs remained viable under the conditions of glyphosate treatment; however, it accumulated in their tissues, resulting in an unsafe food product, since glyphosate has genotoxic, immunotoxic, and potentially, carcinogenic effects (108, 109). Another example of an unpredictable outcome was a genetic modification for the development of a strain-producer of the amino acid L-tryptophan (“Showa Denko K.K.,” Japan), which is used as a dietary supplement (110, 111). An increase in the productivity of the strain unexpectedly led to the contamination of the final product with highly toxic dimerization products of tryptophan precursors, which the proposed purification methods did not remove. A study of the safety of genetically modified soy with reduced linoleic acid content (to improve the properties of soy oil and bring it closer to the quality of olive oil) in mice revealed that its long-term use reduced the likelihood of obesity and the development of insulin resistance; however, it stimulated the development of fatty hepatosis (112).

By themselves, the technologies for the genetic modification of productive animals are not dangerous, especially if we take into account the fact that there are natural mechanisms of horizontal transfer of genetic information even between evolutionarily distant organisms (113–115), which could potentially lead to the emergence of a natural “genetically modified organism.” However, because of the reasons listed above, GMOs or GMO products require thorough comprehensive study as neglect of this rule can, at times, have tragic consequences.

When assessing the safety of food obtained from GMOs, it is possible to use protocols similar to those used in the preclinical study of drugs by assessing acute, chronic, and reproductive toxicity as well as the mutagenic and carcinogenic potential (116). The safety of GMOs for the environment should be considered if there is a risk of “release” of GMOs into the environment. The possibility of transmission and distribution of genes in the wild population should be assessed as well as the “invasive potential” of GMOs to replace natural populations. In some cases, GMOs are designed to replace or modify existing species in nature. This approach is based on gene-drive technology and was proposed to combat the persistence of a parasite (e.g., pathogens of malaria, Dengue fever, Zika) in disease-carrying insects (117, 118).

It has become increasingly clear that genetic technologies and GMOs are the key to solving many problems associated with hunger, epizootics, depletion of natural resources, and environmental pollution (119–121). However, this requires the development of clear legal norms and the creation of an effective independent institute to monitor the use of genomic technologies and the study of the GMOs created.

## 6. Conclusion

Selective breeding for desirable phenotypic traits has been performed in agriculture since the first description of heredity by Gregor Mendel in 1865. This is a time-consuming practice and the desirable phenotype is not guaranteed, due to polygenic traits, complex gene regulation and epigenetics. The vast expansion of the human population following the industrial revolution necessitated large scale changes to agricultural practices in order to sustain and secure the increasing demand for food, medicine and vaccines. It was expected that science would not only contribute, but be at the forefront to develop and implement these changes.

Genome editing technologies have brought significant breakthroughs to routine laboratory work and have revolutionized research by facilitating the study of cells and organisms, on a molecular level. The function of an individual gene, protein or complex could be investigated, by deleting or inserting it into a new host. This in-depth study into the fundamental biology of organisms enabled the application of subsequent changes to the genetic composition of the organism, in order to obtain a highly desirable trait. These traits included the expression of novel proteins in order to transfer novel characteristics to an organism, resulting in a disease resistant or drought tolerant phenotype compared to the parental organism. None of the selective targeting and alteration of specific genes would be possible, if it is not based on a clear and fundamental understanding of the importance and function of the specific gene or protein complex.

The most widely used genome editing technologies include the CRISPR/Cas9 system, chimeric zinc-finger nucleases (ZFN) to create double-strand breaks, and transcription activator-like effector nucleases (TALEN). In recent years, CRISPR/Cas9 has become the preferred, most efficient and cost-effective method for use in editing the genomes of viruses, prokaryotes and eukaryotes. Relying on a single target molecule to guide RNA for DNA sequence recognition, the Cas9 enzyme may be steered toward a specific site with a low risk of off-target effects. This also enables multiplexing in contrast to the other existing gene-targeting technologies.

The applications of CRISPR/Cas9 have far exceeded the potential of only editing genomes, but have additionally been used in diagnostics and third generation sequencing technologies. The ability to timeously and efficiently identify and differentiate between pathogens causing disease, could result in the protection of the livestock by implementing control, prevention or mitigating strategies. The versatility of the implementations of CRISPR-based technologies add value throughout the livestock production chain, including animal production, diagnosis, protection and treatment. The future applications of these technologies would only be limited by the creativity of dedicated scientist.

## Author contributions

All authors listed have made a substantial, direct, and intellectual contribution to the work and approved it for publication.

## Funding

This work was supported by the grant no. 075–15–2021-1054 from the Ministry of Education and Science of Russia to implement objectives of the Federal Scientific and Technical Program for the Development of genetic technologies during 2019–2027.

## Conflict of interest

The authors declare that the research was conducted in the absence of any commercial or financial relationships that could be construed as a potential conflict of interest.

## Publisher’s note

All claims expressed in this article are solely those of the authors and do not necessarily represent those of their affiliated organizations, or those of the publisher, the editors and the reviewers. Any product that may be evaluated in this article, or claim that may be made by its manufacturer, is not guaranteed or endorsed by the publisher.

## References

[1] Bawa AS Anilakumar KR. Genetically modified foods: safety, risks and public concerns-a review. J Food Sci Technol. (2013) 50:1035–46. doi: 10.1007/s13197-012-0899-1, PMID: 24426015 PMC3791249

[2] Karalis DT Karalis T Karalis S Kleisiari AS. Genetically modified products, perspectives and challenges. Cureus. (2020) 12:e7306. doi: 10.7759/cureus.7306, PMID: 32313747 PMC7164548

[3] Ibrahim HMM Ahmad EM Martínez-Medina A Aly MAM. Effective approaches to study the plant-root knot nematode interaction. Plant Physiol Biochem. (2019) 141:332–42. doi: 10.1016/j.plaphy.2019.06.009, PMID: 31207494

[4] Lovett B Bilgo E Diabate A St Leger R. A review of progress toward field application of transgenic mosquitocidal entomopathogenic fungi. Pest Manag Sci. (2019) 75:2316–24. doi: 10.1002/ps.5385, PMID: 30801913

[5] Bishop SC. Genetic resistance to infections in sheep. Vet Microbiol. (2015) 181:2–7. doi: 10.1016/j.vetmic.2015.07.013, PMID: 26260859

[6] Spinoza C Schlechter R Herrera D Torres E Serrano A Medina C. Cisgenesis and intragenesis: new tools for improving crops. Biol Res. (2013) 46:323–31. doi: 10.4067/S0716-97602013000400003, PMID: 24510134

[7] Gupta D Bhattacharjee O Mandal D Sen MK Dey D Dasgupta A. CRISPR-Cas9 system: a new-fangled dawn in gene editing. Life Sci. (2019) 232:116636. doi: 10.1016/j.lfs.2019.116636, PMID: 31295471

[8] Smithies O Gregg RG Boggs SS Koralewski MA Kucherlapati RS. Insertion of DNA sequences into the human chromosomal beta-globin locus by homologous recombination. Nature. (1985) 317:230–4. doi: 10.1038/317230a0, PMID: 2995814

[9] Rouet P Smih F Jasin M. Introduction of double-strand breaks into the genome of mouse cells by expression of a rare-cutting endonuclease. Mol Cell Biol. (1994) 14:8096–106. doi: 10.1128/mcb.14.12.8096-8106.1994, PMID: 7969147 PMC359348

[10] Rouet P Smih F Jasin M. Expression of a site-specific endonuclease stimulates homologous recombination in mammalian cells. Proc Natl Acad Sci U S A. (1994) 91:6064–8. doi: 10.1073/pnas.91.13.6064, PMID: 8016116 PMC44138

[11] Iida S Meyer J Kennedy KE Arber W. A site-specific, conservative recombination system carried by bacteriophage P1. Mapping the recombinase gene cin and the cross-over sites cix for the inversion of the C segment. EMBO J. (1982) 1:1445–53. doi: 10.1002/j.1460-2075.1982.tb01336.x, PMID: 6327269 PMC553230

[12] Gimble FS Thorner J. Homing of a DNA endonuclease gene by meiotic gene conversion in Saccharomyces cerevisiae. Nature. (1992) 357:301–6. doi: 10.1038/357301a0, PMID: 1534148

[13] Hoess R Abremski K Sternberg N. The nature of the interaction of the P1 recombinase Cre with the recombining site loxP. Cold Spring Harb Symp Quant Biol. (1984) 49:761–8. doi: 10.1101/SQB.1984.049.01.086, PMID: 6335689

[14] McLellan MA Rosenthal NA Pinto AR. Cre-loxP-mediated recombination: general principles and experimental considerations. Curr Protoc Mouse Biol. (2017) 7:1–12. doi: 10.1002/cpmo.22, PMID: 28252198

[15] Vetter D Andrews BJ Roberts-Beatty L Sadowski PD. Site-specific recombination of yeast 2-micron DNA in vitro. Proc Natl Acad Sci U S A. (1983) 80:7284–8. doi: 10.1073/pnas.80.23.7284, PMID: 6316354 PMC390039

[16] Schweizer HP. Applications of the *Saccharomyces cerevisiae* Flp-FRT system in bacterial genetics. J Mol Microbiol Biotechnol. (2003) 5:67–77. doi: 10.1159/000069976, PMID: 12736528

[17] Kim H Kim M Im SK Fang S. Mouse Cre-LoxP system: general principles to determine tissue-specific roles of target genes. Lab Anim Res. (2018) 34:147–59. doi: 10.5625/lar.2018.34.4.147, PMID: 30671100 PMC6333611

[18] Miao X. Recent advances in the development of new transgenic animal technology. Cell Mol Life Sci. (2013) 70:815–28. doi: 10.1007/s00018-012-1081-7, PMID: 22833168 PMC11113483

[19] Zou YR Müller W Gu H Rajewsky K. Cre-loxP-mediated gene replacement: a mouse strain producing humanized antibodies. Curr Biol. (1994) 4:1099–103. doi: 10.1016/S0960-9822(00)00248-7, PMID: 7704573

[20] Lui D Burgess SM. Measurement of spatial proximity and accessibility of chromosomal loci in *Saccharomyces cerevisiae* using Cre/loxP site-specific recombination. Methods Mol Biol. (2009) 557:55–63. doi: 10.1007/978-1-59745-527-5_5 19799176 PMC3176630

[21] Martina F Christos E. The revolution of the CRISPR-CAS9 system. United Kingdom: CRISPR Biotech Engineering (2020).

[22] Chevalier BS Stoddard BL. Homing endonucleases: structural and functional insight into the catalysts of intron/intein mobility. Nucleic Acids Res. (2001) 29:3757–74. doi: 10.1093/nar/29.18.3757, PMID: 11557808 PMC55915

[23] Zhao L Bonocora RP Shub DA Stoddard BL. The restriction fold turns to the dark side: a bacterial homing endonuclease with a PD-(D/E)-XK motif. EMBO J. (2007) 26:2432–42. doi: 10.1038/sj.emboj.7601672, PMID: 17410205 PMC1864971

[24] Stoddard BL. Homing endonuclease structure and function. Q Rev Biophys. (2005) 38:49–95. doi: 10.1017/S0033583505004063, PMID: 16336743

[25] Pâques F Duchateau P. Meganucleases and DNA double-strand break-induced recombination: perspectives for gene therapy. Curr Gene Ther. (2007) 7:49–66. doi: 10.2174/156652307779940216, PMID: 17305528

[26] Xu X Hulshoff MS Tan X Zeisberg M Zeisberg EM. CRISPR/Cas derivatives as novel gene modulating tools: possibilities and in vivo applications. Int J Mol Sci. (2020) 21:3038. doi: 10.3390/ijms21093038, PMID: 32344896 PMC7246536

[27] Carroll D. Genome engineering with zinc-finger nucleases. Genetics. (2011) 188:773–82. doi: 10.1534/genetics.111.131433, PMID: 21828278 PMC3176093

[28] Chuang CK Lin WM. Points of view on the tools for Genome/gene editing. Int J Mol Sci. (2021) 22:9872. doi: 10.3390/ijms22189872, PMID: 34576035 PMC8470269

[29] Maeder ML Gersbach CA. Genome-editing Technologies for Gene and Cell Therapy. Mol Ther. (2016) 24:430–46. doi: 10.1038/mt.2016.10, PMID: 26755333 PMC4786923

[30] Joung JK Sander JD. TALENs: a widely applicable technology for targeted genome editing. Nat Rev Mol Cell Biol. (2013) 14:49–55. doi: 10.1038/nrm3486, PMID: 23169466 PMC3547402

[31] Wright DA Li T Yang B Spalding MH. TALEN-mediated genome editing: prospects and perspectives. Biochem J. (2014) 462:15–24. doi: 10.1042/BJ20140295, PMID: 25057889

[32] Mak AN Bradley P Cernadas RA Bogdanove AJ Stoddard BL. The crystal structure of TAL effector PthXo1 bound to its DNA target. Science. (2012) 335:716–9. doi: 10.1126/science.1216211, PMID: 22223736 PMC3427646

[33] Hockemeyer D Jaenisch R. Gene targeting in human pluripotent cells. Cold Spring Harb Symp Quant Biol. (2010) 75:201–9. doi: 10.1101/sqb.2010.75.021 21209393

[34] Mussolino C Morbitzer R Lütge F Dannemann N Lahaye T Cathomen T. A novel TALE nuclease scaffold enables high genome editing activity in combination with low toxicity. Nucleic Acids Res. (2011) 39:9283–93. doi: 10.1093/nar/gkr597, PMID: 21813459 PMC3241638

[35] Doudna JA Charpentier E. The new frontier of genome engineering with CRISPR-Cas9. Science. (2014) 346:1258096. doi: 10.1126/science.1258096 25430774

[36] Jinek M Chylinski K Fonfara I Hauer M Doudna JA Charpentier E. A programmable dual-RNA-guided DNA endonuclease in adaptive bacterial immunity. Science. (2012) 337:816–21. doi: 10.1126/science.1225829, PMID: 22745249 PMC6286148

[37] Ishino Y Krupovic M Forterre P. History of CRISPR-Cas from encounter with a mysterious repeated sequence to Genome editing technology. J Bacteriol. (2018) 200:e00580–17. doi: 10.1128/JB.00580-17 29358495 PMC5847661

[38] Makarova KS Haft DH Barrangou R Brouns SJ Charpentier E Horvath P. Evolution and classification of the CRISPR-Cas systems. Microbiology. (2011) 9:467–77. doi: 10.1038/nrmicro2577, PMID: 21552286 PMC3380444

[39] Nishimasu H Ran FA Hsu PD Konermann S Shehata SI Dohmae N. Crystal structure of Cas9 in complex with guide RNA and target DNA. Cells. (2014) 156:935–49. doi: 10.1016/j.cell.2014.02.001, PMID: 24529477 PMC4139937

[40] Zhang S Shen J Li D Cheng Y. Strategies in the delivery of Cas9 ribonucleoprotein for CRISPR/Cas9 genome editing. Theranostics. (2021) 11:614–48. doi: 10.7150/thno.47007, PMID: 33391496 PMC7738854

[41] Khan SH. Genome-editing technologies: concept, pros, and cons of various Genome-editing techniques and bioethical concerns for clinical application. Mol Ther Nucleic Acids. (2019) 16:326–34. doi: 10.1016/j.omtn.2019.02.027, PMID: 30965277 PMC6454098

[42] Boutros M Ahringer J. The art and design of genetic screens: RNA interference. Nat Rev Genet. (2008) 9:554–66. doi: 10.1038/nrg2364, PMID: 18521077

[43] Van Eenennaam AL. Application of genome editing in farm animals: cattle. Transgenic Res. (2019) 28:93–100. doi: 10.1007/s11248-019-00141-6, PMID: 31321690

[44] Navarro-Serna S Vilarino M Park I Gadea J Ross PJ. Livestock gene editing by one-step embryo manipulation. J Equine Vet Sci. (2020) 89:103025. doi: 10.1016/j.jevs.2020.103025, PMID: 32563448

[45] Wang K Ouyang H Xie Z Yao C Guo N Li M. Efficient generation of myostatin mutations in pigs using the CRISPR/Cas9 system. Sci Rep. (2015) 5:1–11. doi: 10.1038/srep16623 PMC4643223 26564781

[46] Burkard C Lillico SG Reid E Jackson B Mileham AJ Ait-Ali T. Precision engineering for PRRSV resistance in pigs: macrophages from genome edited pigs lacking CD163 SRCR5 domain are fully resistant to both PRRSV genotypes while maintaining biological function. PLoS Pathog. (2017) 13:e1006206. doi: 10.1371/journal.ppat.1006206, PMID: 28231264 PMC5322883

[47] Gao Y Wu H Wang Y Liu X Chen L Li Q. Single Cas9 nickase induced generation of NRAMP1 knockin cattle with reduced off-target effects. Genome Biol. (2017) 18:13. doi: 10.1186/s13059-016-1144-4 28143571 PMC5286826

[48] Zhao Y Wang T Yao L Liu B Teng C Ouyang H. Classical swine fever virus replicated poorly in cells from MxA transgenic pigs. BMC Vet Res. (2016) 12:169. doi: 10.1186/s12917-016-0794-5 27535023 PMC4987965

[49] Xie Z Jiao H Xiao H Jiang Y Liu Z Qi C. Generation of pRSAD2 gene knock-in pig via CRISPR/Cas9 technology. Antivir Res. (2020) 174:104696. doi: 10.1016/j.antiviral.2019.104696, PMID: 31862502

[50] Xie Z Pang D Yuan H Jiao H Lu C Wang K. Genetically modified pigs are protected from classical swine fever virus. PLoS Pathog. (2018) 14:e1007193. doi: 10.1371/journal.ppat.1007193, PMID: 30543715 PMC6292579

[51] Lu C Pang D Li M Yuan H Yu T Huang P. CRISPR/Cas9-mediated hitchhike expression of functional shRNAs at the porcine miR-17-92 cluster. Cells. (2019) 8:113. doi: 10.3390/cells8020113, PMID: 30717310 PMC6406430

[52] Tait-Burkard C Doeschl-Wilson A McGrew MJ Archibald AL Sang HM Houston RD. Livestock 2.0–genome editing for fitter, healthier, and more productive farmed animals. Genome Biol. (2018) 19:204. doi: 10.1186/s13059-018-1583-1 30477539 PMC6258497

[53] Borca MV Berggren KA Ramirez-Medina E Vuono EA Gladue DP. CRISPR/Cas gene editing of a large DNA virus: African swine fever virus. Bio Protoc. (2018) 8:e2978. doi: 10.21769/BioProtoc.2978, PMID: 34395778 PMC8328649

[54] Shen Z Wang G Yang Y Shi J Fang L Li F. A conserved region of nonstructural protein 1 from alphacoronaviruses inhibits host gene expression and is critical for viral virulence. J Biol Chem. (2019) 294:13606–18. doi: 10.1074/jbc.RA119.009713, PMID: 31350335 PMC6746460

[55] Yuan M Gao X Chard LS Ali Z Ahmed J Li Y. A marker-free system for highly efficient construction of vaccinia virus vectors using CRISPR Cas9. Mol Ther Methods Clin Dev. (2015) 2:15035. doi: 10.1038/mtm.2015.35, PMID: 26417609 PMC4571730

[56] Di Gioia C Yuan M Wang Y. Vaccinia virus Genome editing using CRISPR. Methods Mol Biol. (2019) 2023:109–17. doi: 10.1007/978-1-4939-9593-6_6 31240673

[57] Chou Y-Y Krupp A Kaynor C Gaudin R Ma M Cahir-McFarland E. Inhibition of JCPyV infection mediated by targeted viral genome editing using CRISPR/Cas9. Sci Rep. (2016) 6:36921. doi: 10.1038/srep36921 27841295 PMC5107994

[58] Hübner A Petersen B Keil GM Niemann H Mettenleiter TC Fuchs W. Efficient inhibition of African swine fever virus replication by CRISPR/Cas9 targeting of the viral p30 gene (CP204L). Sci Rep. (2018) 8:1449. doi: 10.1038/s41598-018-19626-1 29362418 PMC5780455

[59] Abkallo HM Svitek N Oduor B Awino E Henson SP Oyola SO. Rapid CRISPR/Cas9 editing of genotype IX African swine fever virus circulating in eastern and Central Africa. Front Genet. (2021) 12:733674. doi: 10.3389/fgene.2021.733674, PMID: 34527025 PMC8435729

[60] Borca MV Holinka LG Berggren KA Gladue DP. CRISPR-Cas9, a tool to efficiently increase the development of recombinant African swine fever viruses. Sci Rep. (2018) 8:3154. doi: 10.1038/s41598-018-21575-8 29453406 PMC5816594

[61] Pan Q Wang J Gao Y Cui H Liu C Qi X. The natural large genomic deletion is unrelated to the increased virulence of the novel genotype fowl adenovirus 4 recently emerged in China. Viruses. (2018) 10:494. doi: 10.3390/v10090494, PMID: 30217040 PMC6165077

[62] Liang X Sun L Yu T Pan Y Wang D Hu X. A CRISPR/Cas9 and Cre/lox system-based express vaccine development strategy against re-emerging pseudorabies virus. Sci Rep. (2016) 6:19176. doi: 10.1038/srep19176 26777545 PMC4726036

[63] Xu A Qin C Lang Y Wang M Lin M Li C. A simple and rapid approach to manipulate pseudorabies virus genome by CRISPR/Cas9 system. Biotechnol Lett. (2015) 37:1265–72. doi: 10.1007/s10529-015-1796-2, PMID: 25724716

[64] Hagag IT Wight DJ Bartsch D Sid H Jordan I Bertzbach LD. Abrogation of Marek’s disease virus replication using CRISPR/Cas9. Sci Rep. (2020) 10:10919. doi: 10.1038/s41598-020-67951-1 32616820 PMC7331644

[65] Price AA Sampson TR Ratner HK Grakoui A Weiss DS. Cas9-mediated targeting of viral RNA in eukaryotic cells. Proc Natl Acad Sci U S A. (2015) 112:6164–9. doi: 10.1073/pnas.1422340112, PMID: 25918406 PMC4434742

[66] Li H Wang S Dong X Li Q Li M Li J. CRISPR-Cas13a cleavage of dengue virus NS3 gene efficiently inhibits viral replication. Mol Ther Nucleic Acids. (2020) 19:1460–9. doi: 10.1016/j.omtn.2020.01.028, PMID: 32160714 PMC7056623

[67] Cui J Techakriengkrai N Nedumpun T Suradhat S. Abrogation of PRRSV infectivity by CRISPR-Cas13b-mediated viral RNA cleavage in mammalian cells. Sci Rep. (2020) 10:1–13. doi: 10.1038/s41598-020-66775-3 32541822 PMC7295971

[68] Smith GL Moss B. Infectious poxvirus vectors have capacity for at least 25,000 base pairs of foreign DNA. Gene. (1983) 25:21–8. doi: 10.1016/0378-1119(83)90163-4, PMID: 6229451

[69] Moss B Carroll MW Wyatt LS Bennink JR Hirsch VM Goldstein S. Host range restricted, non-replicating vaccinia virus vectors as vaccine candidates. Adv Exp Med Biol. (1996) 397:7–13. doi: 10.1007/978-1-4899-1382-1_2 8718576 PMC2562214

[70] Werden SJ Rahman MM McFadden G. Poxvirus host range genes. Adv Virus Res. (2008) 71:135–71. doi: 10.1016/S0065-3527(08)00003-1 18585528

[71] Gudmundsdotter L Nilsson C Brave A Hejdeman B Earl P Moss B. Recombinant modified vaccinia Ankara (MVA) effectively boosts DNA-primed HIV-specific immune responses in humans despite pre-existing vaccinia immunity. Vaccine. (2009) 27:4468–74. doi: 10.1016/j.vaccine.2009.05.018, PMID: 19450644 PMC4788966

[72] Smith GL Mackett M Moss B. Infectious vaccinia virus recombinants that express hepatitis B virus surface antigen. Nature. (1983) 302:490–5. doi: 10.1038/302490a0, PMID: 6835382

[73] Draper SJ Heeney JL. Viruses as vaccine vectors for infectious diseases and cancer. Nat Rev Microbiol. (2010) 8:62–73. doi: 10.1038/nrmicro2240, PMID: 19966816

[74] Ramirez JC Gherardi MM Esteban M. Biology of attenuated modified vaccinia virus Ankara recombinant vector in mice: virus fate and activation of B- and T-cell immune responses in comparison with the Western reserve strain and advantages as a vaccine. J Virol. (2000) 74:923–33. doi: 10.1128/JVI.74.2.923-933.2000, PMID: 10623755 PMC111613

[75] Garcia-Arriaza J Esteban M. Enhancing poxvirus vectors vaccine immunogenicity. Hum Vaccin Immunother. (2014) 10:2235–44. doi: 10.4161/hv.28974, PMID: 25424927 PMC4896794

[76] Yang DK Kim HH Lee KW Song JY. The present and future of rabies vaccine in animals. Clin Exp Vaccine Res. (2013) 2:19–25. doi: 10.7774/cevr.2013.2.1.19, PMID: 23596586 PMC3623496

[77] Wyatt LS Earl PL Moss B. Generation of recombinant vaccinia viruses. Curr Protoc Mol Biol. (2017) 117:16.17.11–8. doi: 10.1002/cpmb.32 28060405

[78] Merchlinsky M Moss B. Introduction of foreign DNA into the vaccinia virus genome by in vitro ligation: recombination-independent selectable cloning vectors. Virology. (1992) 190:522–6. doi: 10.1016/0042-6822(92)91246-Q, PMID: 1529553

[79] Domi A Moss B. Cloning the vaccinia virus genome as a bacterial artificial chromosome in Escherichia coli and recovery of infectious virus in mammalian cells. Proc Natl Acad Sci U S A. (2002) 99:12415–20. doi: 10.1073/pnas.192420599, PMID: 12196634 PMC129459

[80] Paszkowski P Noyce RS Evans DH. Live-cell imaging of vaccinia virus recombination. PLoS Pathog. (2016) 12:e1005824. doi: 10.1371/journal.ppat.1005824, PMID: 27525721 PMC4985154

[81] Ball LA. High-frequency homologous recombination in vaccinia virus DNA. J Virol. (1987) 61:1788–95. doi: 10.1128/jvi.61.6.1788-1795.1987, PMID: 3573150 PMC254181

[82] Yuan M Wang P Chard LS Lemoine NR Wang Y. A simple and efficient approach to construct mutant vaccinia virus vectors. J Vis Exp. (2016) 116:54171. doi: 10.3791/54171 PMC5226142 27842362

[83] Wyatt LS Belyakov IM Earl PL Berzofsky JA Moss B. Enhanced cell surface expression, immunogenicity and genetic stability resulting from a spontaneous truncation of HIV env expressed by a recombinant MVA. Virology. (2008) 372:260–72. doi: 10.1016/j.virol.2007.10.033 18048074 PMC2289778

[84] McNeel DG Chen Y-H Gulley JL Dwyer AJ Madan RA Carducci MA. Randomized phase II trial of docetaxel with or without PSA-TRICOM vaccine in patients with castrate-resistant metastatic prostate cancer: a trial of the ECOG-ACRIN cancer research group (E1809). Hum Vaccin Immunother. (2015) 11:2469–74. doi: 10.1080/21645515.2015.1062190, PMID: 26111351 PMC4635940

[85] Gulley JL Madan RA Tsang KY Jochems C Marté JL Farsaci B. Immune impact induced by PROSTVAC (PSA-TRICOM), a therapeutic vaccine for prostate cancer. Cancer Immunol Res. (2014) 2:133–41. doi: 10.1158/2326-6066.CIR-13-0108, PMID: 24778277 PMC4004961

[86] Arlen PM Gulley JL Parker C Skarupa L Pazdur M Panicali D. A randomized phase II study of concurrent docetaxel plus vaccine versus vaccine alone in metastatic androgen-independent prostate cancer. Clin Cancer Res. (2006) 12:1260–9. doi: 10.1158/1078-0432.CCR-05-2059, PMID: 16489082 PMC1526707

[87] Kaufman HL Deraffele G Mitcham J Moroziewicz D Cohen SM Hurst-Wicker KS. Targeting the local tumor microenvironment with vaccinia virus expressing B7.1 for the treatment of melanoma. J Clin Investig. (2005) 115:1903–12. doi: 10.1172/JCI24624, PMID: 15937544 PMC1142116

[88] Okoli AS Asare N Gjøen T Klein J Ytrehus B. Knowledge Base for the assessment of environmental risks by the use of genetically modified virus-vectored vaccines for domesticated animals; scientific opinion of the panel on microbial ecology of the Norwegian scientific Committee for Food Safety (VKM). Oslo, Norway: Norwegian Scientific Committee for Food Safety (2016).

[89] Fehlner-Gardiner C Rudd R Donovan D Slate D Kempf L Badcock J. Comparing ONRAB(R) and RABORAL V-RG(R) oral rabies vaccine field performance in raccoons and striped skunks. J Wildl Dis. (2012) 48:157–67. doi: 10.7589/0090-3558-48.1.157, PMID: 22247384

[90] Baggen J Persoons L Vanstreels E Jansen S Van Looveren D Boeckx B. Genome-wide CRISPR screening identifies TMEM106B as a proviral host factor for SARS-CoV-2. Nat Genet. (2021) 53:435–44. doi: 10.1038/s41588-021-00805-2, PMID: 33686287

[91] Hultquist JF Hiatt J Schumann K McGregor MJ Roth TL Haas P. CRISPR–Cas9genome engineering of primary CD4+ T cells for the interrogation of HIV–host factor interactions. Nat Protoc. (2019) 14:1–27. doi: 10.1038/s41596-018-0069-7, PMID: 30559373 PMC6637941

[92] Zhang R Kim AS Fox JM Nair S Basore K Klimstra WB. Mxra8 is a receptor for multiple arthritogenic alphaviruses. Nature. (2018) 557:570–4. doi: 10.1038/s41586-018-0121-3, PMID: 29769725 PMC5970976

[93] Shin HJ Ku KB Kim S Kim HS Kim YS Kim BT. A crucial role of ACBD3 required for coxsackievirus infection in animal model developed by AAV-mediated CRISPR Genome editing technique. Viruses. (2021) 13:237. doi: 10.3390/v13020237, PMID: 33546322 PMC7913485

[94] Ma H Kim AS Kafai NM Earnest JT Shah AP Case JB. LDLRAD3 is a receptor for Venezuelan equine encephalitis virus. Nat Cell Biol. (2020) 588:308–14. doi: 10.1038/s41586-020-2915-3 PMC7769003 33208938

[95] Sharon DM Nesdoly S Yang HJ Gélinas J-F Xia Y Ansorge S. A pooled genome-wide screening strategy to identify and rank influenza host restriction factors in cell-based vaccine production platforms. Sci Rep. (2020) 10:1–15. doi: 10.1038/s41598-020-68934-y 32699298 PMC7376217

[96] Forst CV Zhou B Wang M Chou T-W Mason G Song W-M. Integrative gene network analysis identifies key signatures, intrinsic networks and host factors for influenza virus a infections. NPJ Syst Biol Appl. (2017) 3:35. doi: 10.1038/s41540-017-0036-x 29214055 PMC5712526

[97] Kim TH Kern C Zhou H. Knockout of IRF7 highlights its modulator function of host response against avian InfluenzaVirus and the involvement of MAPK and TOR Signaling pathways in chicken. Genes. (2020) 11:385. doi: 10.3390/genes11040385, PMID: 32252379 PMC7230310

[98] Cui J Morgan D Cheng DH Foo SL Yap GLR Ampomah PB. RNA-sequencing-based transcriptomic analysis reveals a role for annexin-A1 in classical and in-fluenza a virus-induced autophagy. Cells. (2020) 9:1399. doi: 10.3390/cells9061399, PMID: 32512864 PMC7349256

[99] Tran V Ledwith MP Thamamongood T Higgins CA Tripathi S Chang MW. Influenza virus repurposes the antiviral protein IFIT2 to promote translation of viral mRNAs. Nat Microbiol. (2020) 5:1490–503. doi: 10.1038/s41564-020-0778-x, PMID: 32839537 PMC7677226

[100] Gadalla MR Morrison E Serebryakova MV Han X Wolff T Freund C. NS1-mediated upregulation of ZDHHC22 acyltransferase in influenza a virus infected cells. Cell Microbiol. (2021):e13322. doi: 10.1111/cmi.13322, PMID: 33629465

[101] Ricroch AE Hénard-Damave MC. Next biotech plants: new traits, crops, developers and technologies for addressing global challenges. Crit Rev Biotechnol. (2016) 36:675–90. doi: 10.3109/07388551.2015.1004521, PMID: 25641327

[102] Álvarez-Alfageme F Devos Y Camargo AM Arpaia S Messéan A. Managing resistance evolution to transgenic Bt maize in corn borers in Spain. Crit Rev Biotechnol. (2022) 42:201–19. doi: 10.1080/07388551.2021.1931018, PMID: 34154477

[103] Hu W Zheng H Li Q Wang Y Liu X Hu X. shRNA transgenic swine display resistance to infection with the foot-and-mouth disease virus. Sci Rep. (2021) 11:16377. doi: 10.1038/s41598-021-95853-3 34385528 PMC8361160

[104] Jaganathan D Ramasamy K Sellamuthu G Jayabalan S Venkataraman G. CRISPR for crop improvement: an update review. Front Plant Sci. (2018) 9:985. doi: 10.3389/fpls.2018.00985 30065734 PMC6056666

[105] De Santis B Stockhofe N Wal JM Weesendorp E Lallès JP van Dijk J. Case studies on genetically modified organisms (GMOs): potential risk scenarios and associated health indicators. Food Chem Toxicol. (2018) 117:36–65. doi: 10.1016/j.fct.2017.08.033, PMID: 28859885

[106] Pott A Otto M Schulz R. Impact of genetically modified organisms on aquatic environments: review of available data for the risk assessment. Sci Total Environ. (2018) 635:687–98. doi: 10.1016/j.scitotenv.2018.04.013 29680759

[107] Bøhn T Cuhra M Traavik T Sanden M Fagan J Primicerio R. Compositional differences in soybeans on the market: glyphosate accumulates in roundup ready GM soybeans. Food Chem. (2014) 153:207–15. doi: 10.1016/j.foodchem.2013.12.054, PMID: 24491722

[108] Andreotti G Koutros S Hofmann JN Sandler DP Lubin JH Lynch CF. Glyphosate use and cancer incidence in the agricultural health study. J Natl Cancer Inst. (2018) 110:509–16. doi: 10.1093/jnci/djx233 29136183 PMC6279255

[109] Peillex C Pelletier M. The impact and toxicity of glyphosate and glyphosate-based herbicides on health and immunity. J Immunotoxicol. (2020) 17:163–74. doi: 10.1080/1547691X.2020.1804492, PMID: 32897110

[110] Roberts L. L-tryptophan puzzle takes new twist. Science. (1990) 249:988. doi: 10.1126/science.2396102, PMID: 2396102

[111] Mann LRB Straton B Christ WE. *The thalidomide of genetic engineering*. Biosafety Information Centre. (2005).

[112] Deol P Fahrmann J Yang J Evans JR Rizo A Grapov D. Omega-6 and omega-3 oxylipins are implicated in soybean oil-induced obesity in mice. Sci Rep. (2017) 7:12488. doi: 10.1038/s41598-017-12624-9 28970503 PMC5624939

[113] Guo M Ye J Gao D Xu N Yang J. Agrobacterium-mediated horizontal gene transfer: mechanism, biotechnological application, potential risk and forestalling strategy. Biotechnol Adv. (2019) 37:259–70. doi: 10.1016/j.biotechadv.2018.12.008, PMID: 30579929

[114] Huang W Tsai L Li Y Hua N Sun C Wei C. Widespread of horizontal gene transfer in the human genome. BMC Genomics. (2017) 18:274. doi: 10.1186/s12864-017-3649-y 28376762 PMC5379729

[115] Graham LA Davies PL. Horizontal gene transfer in vertebrates: a fishy Tale. Trends Genet. (2021) 37:501–3. doi: 10.1016/j.tig.2021.02.006 33714557

[116] EFSA GMO Panel Working Group on Animal Feeding Trials. Safety and nutritional assessment of GM plants and derived food and feed: the role of animal feeding trials. Food Chem Toxicol. (2008) 46:70. doi: 10.1016/j.fct.2008.02.008 18328408

[117] Hammond A Galizi R Kyrou K Simoni A Siniscalchi C Katsanos D. A CRISPR-Cas9 gene drive system targeting female reproduction in the malaria mosquito vector *Anopheles gambiae*. Nat Biotechnol. (2016) 34:78–83. doi: 10.1038/nbt.3439, PMID: 26641531 PMC4913862

[118] Nateghi Rostami M. CRISPR/Cas9 gene drive technology to control transmission of vector-borne parasitic infections. Parasite Immunol. (2020) 42:e12762. doi: 10.1111/pim.12762, PMID: 32497313

[119] Okoli AS Blix T Myhr AI Xu W Xu X. Sustainable use of CRISPR/Cas in fish aquaculture: the biosafety perspective. Transgenic Res. (2022) 31:1–21. doi: 10.1007/s11248-021-00274-7, PMID: 34304349 PMC8821480

[120] Carter BE Conn CC Wiles JR. Concern about hunger may increase receptivity to GMOs. Trends Plant Sci. (2016) 21:539–41. doi: 10.1016/j.tplants.2016.05.003, PMID: 27246454

[121] Scott SE Inbar Y Wirz CD Brossard D Rozin P. An overview of attitudes toward genetically engineered food. Annu Rev Nutr. (2018) 38:459–79. doi: 10.1146/annurev-nutr-071715-051223, PMID: 29801421

