# Supplementary material for: Post-genomic era in agriculture and veterinary science: successful and proposed application of genetic targeting technologies
Source: Front Vet Sci. 2023 Aug 3;10:1180621. doi: 10.3389/fvets.2023.1180621 (PMC10434572; doi:10.3389/fvets.2023.1180621)
Supplement: Supplementary file 1 [file Table_1.docx]

**Supplementary Material:**

Supplementary table 1. Application of CRISPR/Cas for bacterial genome modification

| **Bacteria** | **Technology used** | **Application** |
| --- | --- | --- |
| **Escherichia**  [122-132] | | |
| *E. coli* | Cas9-Mediated genome editing | Production of uridine, adipic acid, β-carotene and isopropanol |
|  | Cas12a-Mediated genome editing | Complementary to biotechnology |
|  | dCas9-mediated CRISPRa | Complementary to biotechnology |
|  | dCas9-mediated CRISPRi | Production of lycopene, isoprene, 4-hydroxybutyrate, malic acid, butanol, malonyl-CoA, mevalonic acid, naringenin |
|  | dCas12a-mediated CRISPRi | Complementary to biotechnology |
|  | DNA editing | Complementary to biotechnology |
|  | RNA editing | Complementary to biotechnology |
|  | RNA cleavage | Complementary to biotechnology |
| **Cyanobacteria**  [133-135] | | |
| *Synechococcus* | Cas12a-Mediated genome editing | Complementary to biotechnology |
| *Synechocystis* | Cas12a-Mediated genome editing | Complementary to biotechnology |
| *Anabaena* | Cas12a-Mediated genome editing | Complementary to biotechnology |
| *S. elongatus* | Cas9-Mediated genome editing | Complementary to biotechnology |
| *Synechocystis* | Cas9-Mediated genome editing | Complementary to biotechnology |
| **Streptomyces**  [136-142] | | |
| *S. coelicolor* | Cas9-Mediated genome editing | Production of secondary metabolic products |
| *S. ablus* | Cas9-Mediated genome editing | Activation of silent BGCs |
| *S. viridochromogenes* | Cas9-Mediated genome editing | Activation of silent BGCs |
| *S. lividans* | Cas9-Mediated genome editing | Activation of silent BGCs |
| *S. coelicolor* | dCas12a-mediated CRISPRi | Производство вторичных продуктов метаболизма Production of secondary metabolic products |
| *S. hygroscopicus* | Cas12a-Mediated genome editing | Production of 5-oxomylbemycin |
| *S. rimosus* | Cas9-Mediated genome editing | Production of oxytetracycline |
| *S. coelicolor* | dCas9-mediated CRISPRi | Production of secondary metabolic products |
| *S. venezuelae* | Cas9-Mediated genome editing | Activation of silent BGCs |
| **Lactic acid bacteria**  [143, 144] | | |
| L. reuteri | Cas9-Mediated genome editing | Complementary to biotechnology |
| L. casei | Cas9-Mediated genome editing | Complementary to biotechnology |
| **Clostridium**  [145-158] | | |
| *C. beijerinckii* | Cas9-Mediated genome editing | Complementary to biotechnology |
| *C. saccharoperbutylacetonicum* | Cas9-Mediated genome editing | Buthanol production |
| *C. acetobutylicum* | dCas9-mediated CRISPRi | Complementary to biotechnology |
| *C. beijerinckii* | nCas9-Mediated genome editing, dCas9-mediated CRISPRi | Complementary to biotechnology |
| *C. ljungdahlii* | Cas9-Mediated genome editing | Production of ethanol from synthetic fuel |
| *C. tyrobutyricum* | Cas9-Mediated genome editing | Buthanol production |
| *C. pasteurianum* | Cas9-Mediated genome editing | Production of butanol from glycerine |
| *C. difficile* | Cas12a-Mediated genome editing | Complementary to biotechnology |
| *C. cellulolyticum* | nCas9-Mediated genome editing | Biofuel production from lignin |
| *C. acetobutylicum* | dCas9-mediated CRISPRi | Reducing the inhibitory effect of metabolic products |
| *C. beijerinckii* | dCas9-mediated CRISPRi | Complementary to biotechnology |
| *C. cellulovorans* | dCas9-mediated CRISPRi | Production of acetone, butanol and ethanol |
| **Corynebacterium**  [159-167] | | |
| *C. glutamicum* | Cas12a-Mediated genome editing | Complementary to biotechnology |
|  | Cas9-Mediated genome editing | Production of γ-aminobutyrate and 1,2-propanediol |
|  | Cas9 и nCas9-Mediated genome editing | Glutamine production |
|  | dCas9-mediated CRISPRi | Production of L-lysine, L-glutamate and homobutyrate |
| **Bacillus**  [168-173] | | |
| *B. subtilis* | Cas9-Mediated genome editing | Production of L-valine and β-cyclodextrin glycosyltransferase |
|  | dCas9-mediated CRISPRi | Production of hyaluronic acid and N-acetylglucosamine |
| *B. smithii* | Cas9-Mediated genome editing | Complementary to biotechnology |
| **Pathogenic bacteria**  [174-182] | | |
| *S. aureus* | DNA editing | Complementary to biotechnology |
| *S. aureus* | Cas9-Mediated genome editing | Complementary to biotechnology |
| *S. aureus* | dCas9-mediated CRISPRi | Complementary to biotechnology |
| *M. tuberculosis* | dCas9-mediated CRISPRi | Complementary to biotechnology |
| *P. aeruginosa* | dCas9-mediated CRISPRi | Complementary to biotechnology |
| *Klebsiella* | Cas9-Mediated genome editing | Complementary to biotechnology |
| *K. pneumoniae* | dCas9-mediated CRISPRi | Complementary to biotechnology |
| *Y. pestis* | Cas12a-Mediated genome editing | Complementary to biotechnology |

**Supplementary References:**

122. Jiang, W.; Bikard, D.; Cox, D.; Zhang, F.; Marraffini, L.A. RNA-guided editing of bacterial genomes using CRISPR-Cas systems. Nat. Biotechnol. 2013, 31, 233–239.

123. Li, Y.; Lin, Z.; Huang, C.; Zhang, Y.; Wang, Z. Metabolic engineering of Escherichia coli using CRISPR – Cas9 meditated genome editing. Metab. Eng. 2015, 31, 13–21.

124. Liang, L.; Liu, R.; Garst, A.D.; Lee, T.; Sànchez, V.; Beckham, T.; Gill, R.T. CRISPR enabled trackable genome engineering for isopropanol production in Escherichia coli. Metab. Eng. 2017, 41, 1–10.

125. Wu, H.; Li, Y.; Ma, Q.; Li, Q.; Jia, Z.; Yang, B.; Xu, Q.; Fan, X.; Zhang, C.; Chen N. Metabolic engineering of Escherichia coli for high-yield uridine production. Metab. Eng. 2018, 49, 248–256.

126. Zhao, M.; Huang, D.; Zhang, X.; Koffas, M.A.G.; Zhou, J.; Deng, Y. Metabolic engineering of Escherichia coli for producing adipic acid through the reverse adipate-degradation pathway. Metab. Eng. 2018, 47, 254–262.

127. Yan, M.Y.; Yan, H.Q.; Ren, G.X.; Zhao, J.P.; Guo, X.P.; Sun, Y.C. CRISPR-Cas12a-Assisted recombineering in bacteria. Appl. Environ. Microbiol. 2017, 83.

128. Keun, S.; Hwan, G.; Seong, W.; Kim, H.; Kim, S.; Lee, D.; Lee, S. CRISPR interference-guided balancing of a biosynthetic mevalonate pathway increases terpenoid production. Metab. Eng. 2016, 38, 228–240.

129. Lv, L.; Ren, Y.; Chen, J.; Wu, Q.; Chen, G. Application of CRISPRi for prokaryotic metabolic engineering involving multiple genes, a case study: Controllable P (3HB- co -4HB ) biosynthesis. Metab. Eng. 2015, 29, 160–168.

130. Gao C., Wang S., Hu G., Guo L., Chen X. Engineering Escherichia coli for malate production by integrating modular pathway characterization with CRISPRi-guided multiplexed metabolic tuning. Biotechnol Bioeng. 2017;115:661–672.

131. Kim S.K., Seong W., Han G.H., Lee D.H., Lee S.G. CRISPR interference - guided multiplex repression of endogenous competing pathway genes for redirecting metabolic flux in Escherichia coli. Microb Cell Fact. 2017:1–15.

132. Chu L.L., Dhakal D., Shin H.J., Jung H.J., Yamaguchi T., Sohng J.K. Metabolic engineering of Escherichia coli for enhanced production of naringenin 7-sulfate and its biological activities. Front Microbiol. 2018;9:1671.

133. Ungerer J., Pakrasi H.B. Cpf1 is a versatile tool for CRISPR genome editing across diverse species of cyanobacteria. Sci Rep. 2016;6:39681.

134. Griese M., Lange C., Soppa J. Ploidy in cyanobacteria. FEMS Microbiol Lett. 2011;323:124131.

135. Li H., Shen C.R., Huang C.H., Sung L.Y., Wu M.Y., Hu Y.C. CRISPR-Cas9 for the genome engineering of cyanobacteria and succinate production. Metab Eng. 2016;38:293–302.

136. Huang H., Zheng G., Jiang W., Hu H., Lu Y. One-step high-efficiency CRISPR/Cas9-mediated genome editing in Streptomyces. Acta Biochim Biophys Sin (Shanghai) 2015;47:231–243.

137. Cobb R.E., Wang Y., Zhao H. High-efficiency multiplex genome editing of Streptomyces species using an engineered CRISPR/Cas system. ACS Synth Biol. 2015;4:723–728.

138. Zeng H., Wen S., Xu W., He Z., Zhai G., Liu Y., Deng Z., Sun Y. Highly efficient editing of the actinorhodin polyketide chain length factor gene in Streptomyces coelicolor M145 using CRISPR/Cas9-CodA(sm) combined system. Appl Microbiol Biotechnol. 2015;99:10575–10585.

139. Li L., Wei K., Zheng G., Liu X., Chen S., Jiang W., Lu Y. CRISPR-Cpf1 assisted multiplex genome editing and transcriptional repression in Streptomyces. Appl Environ Microbiol. 2018 pii: e00827-18.

140. Jia H., Zhang L., Wang T., Han J., Tang H. Development of a CRISPR/Cas9-mediated gene-editing tool in Streptomyces rimosus. Microbiology. 2017;163:1148–1155.

141. Tong Y., Charusanti P., Zhang L., Weber T., Lee S.Y. CRISPR-Cas9 based engineering of actinomycetal genomes. ACS Synth Biol. 2015;4:1020–1029.

142. Zhang M.M., Wong F.T., Wang Y., Luo S., Lim Y.H., Heng E., Yeo W.L., Cobb R.E., Enghiad B., Ang E.L. CRISPR-Cas9 strategy for activation of silent Streptomyces biosynthetic gene clusters. Nat Chem Biol. 2017;13:607–611.

143. Oh J.H., van Pijkeren J.P. CRISPR-Cas9-assisted recombineering in Lactobacillus reuteri. Nucleic Acids Res. 2014;42:e131.

144. Song X., Huang H., Xiong Z., Ai L., Yang S. CRISPR-Cas9(D10A) nickase-assisted genome editing in Lactobacillus casei. Appl Environ Microbiol. 2017;83

145. Wang Y., Zhang Z.T., Seo S.O., Choi K., Lu T., Jin Y.S., Blaschek H.P. Markerless chromosomal gene deletion in Clostridium beijerinckii using CRISPR/Cas9 system. J Biotechnol. 2015;200:1–5.

146. Wang Y., Zhang Z.T., Seo S.O., Lynn P., Lu T., Jin Y.S., Blaschek H.P. Bacterial genome editing with CRISPR-Cas9: deletion, integration, single nucleotide modification, and desirable “clean” mutant selection in Clostridium beijerinckii as an example. ACS Synth Biol. 2016;5:721–732.

147. Wang S., Dong S., Wang P., Tao Y., Wang Y. Genome editing in Clostridium saccharoperbutylacetonicum N1-4 with the CRISPR-Cas9 system. Appl Environ Microbiol. 2017;83

148. Li Q., Chen J., Minton N.P., Zhang Y., Wen Z., Liu J., Yang H., Zeng Z., Ren X., Yang J. CRISPR-based genome editing and expression control systems in Clostridium acetobutylicum and Clostridium beijerinckii. Biotechnol J. 2016;11:961–972.

149. Nagaraju S., Davies N.K., Walker D.J., Kopke M., Simpson S.D. Genome editing of Clostridium autoethanogenum using CRISPR/Cas9. Biotechnol Biofuels. 2016;9:219.

150. Huang H., Chai C., Li N., Rowe P., Minton N.P., Yang S., Jiang W., Gu Y. CRISPR/Cas9-Based efficient genome editing in Clostridium ljungdahlii, an autotrophic gas-fermenting bacterium. ACS Synth Biol. 2016;5:1355–1361.

151. Zhang J., Zong W., Hong W., Zhang Z.T., Wang Y. Exploiting endogenous CRISPR-Cas system for multiplex genome editing in Clostridium tyrobutyricum and engineer the strain for high-level butanol production. Metab Eng. 2018;47:49–59.

152. Pyne M.E., Bruder M.R., Moo-Young M., Chung D.A., Chou C.P. Harnessing heterologous and endogenous CRISPR-Cas machineries for efficient markerless genome editing in Clostridium. Sci Rep. 2016;6:25666.

153. Hong W., Zhang J., Cui G., Wang L., Wang Y. Multiplexed CRISPR-Cpf1-mediated genome editing in Clostridium difficile toward the understanding of pathogenesis of C. difficile infection. ACS Synth Biol. 2018;7:1588–1600.

154. Xu T., Li Y., Shi Z., Hemme C.L., Zhu Y., Van Nostrand J.D., He Z., Zhou J. Efficient genome editing in Clostridium cellulolyticum via CRISPR-Cas9 nickase. Appl Environ Microbiol. 2015;81:4423–4431.

155. Xu T., Li Y., He Z., Van Nostrand J.D., Zhou J. Cas9 nickase-assisted RNA repression enables stable and efficient manipulation of essential metabolic genes in Clostridium cellulolyticum. Front Microbiol. 2017;8:1744.

156. Bruder M.R., Pyne M.E., Moo-Young M., Chung D.A., Chou C.P. Extending CRISPR-Cas9 technology from genome editing to transcriptional engineering in the genus Clostridium. Appl Environ Microbiol. 2016;82:6109–6119.

157. Wang Y., Zhang Z.T., Seo S.O., Lynn P., Lu T., Jin Y.S., Blaschek H.P. Gene transcription repression in Clostridium beijerinckii using CRISPR-dCas9. Biotechnol Bioeng. 2016;113:2739–2743.

158. Wen Z., Minton N.P., Zhang Y., Li Q., Liu J., Jiang Y., Yang S. Enhanced solvent production by metabolic engineering of a twin-clostridial consortium. Metab Eng. 2017;39:38–48.

159. Jiang Y., Qian F., Yang J., Liu Y., Dong F., Xu C., Sun B., Chen B., Xu X., Li Y. CRISPR-Cpf1 assisted genome editing of Corynebacterium glutamicum. Nat Commun. 2017;8:15179.

160. Liu J., Wang Y., Lu Y., Zheng P., Sun J., Ma Y. Development of a CRISPR/Cas9 genome editing toolbox for Corynebacterium glutamicum. Microb Cell Fact. 2017;16:205.

161. Peng F., Wang X., Sun Y., Dong G., Yang Y., Liu X., Bai Z. Efficient gene editing in Corynebacterium glutamicum using the CRISPR/Cas9 system. Microb Cell Fact. 2017;16:201.

162. Cho J.S., Choi K.R., Prabowo C.P.S., Shin J.H., Yang D., Jang J., Lee S.Y. CRISPR/Cas9-coupled recombineering for metabolic engineering of Corynebacterium glutamicum. Metab Eng. 2017;42:157–167.

163. Wang B., Hu Q., Zhang Y., Shi R., Chai X., Liu Z., Shang X., Wen T. A RecET-assisted CRISPR-Cas9 genome editing in Corynebacterium glutamicum. Microb Cell Fact. 2018;17:63.

164. Wang Y., Liu Y., Liu J., Guo Y., Fan L., Ni X., Zheng X., Wang M., Zheng P., Sun J. MACBETH: multiplex automated Corynebacterium glutamicum base editing method. Metab Eng. 2018;47:200–210.

165. Cleto S., Jensen J.V., Wendisch V.F., Lu T.K. Corynebacterium glutamicum metabolic engineering with CRISPR interference (CRISPRi) ACS Synth Biol. 2016;5:375–385.

166. Park J., Shin H., Lee S.M., Um Y., Woo H.M. RNA-guided single/double gene repressions in Corynebacterium glutamicum using an efficient CRISPR interference and its application to industrial strain. Microb Cell Fact. 2018;17:4.

167. Yoon J., Woo H.M. CRISPR interference-mediated metabolic engineering of Corynebacterium glutamicum for homo-butyrate production. Biotechnol Bioeng. 2018;115:2067–2074.

168. Westbrook A.W., Moo-Young M., Chou C.P. Development of a CRISPR-Cas9 tool kit for Comprehensive engineering of Bacillus subtilis. Appl Environ Microbiol. 2016;82:4876–4895.

169. Westbrook A.W., Ren X., Moo-Young M., Chou C.P. Metabolic engineering of Bacillus subtilis for L-valine overproduction. Biotechnol Bioeng. 2018

170. Westbrook A.W., Ren X., Oh J., Moo-Young M., Chou C.P. Metabolic engineering to enhance heterologous production of hyaluronic acid in Bacillus subtilis. Metab Eng. 2018;47:401–413.

171. Wu Y., Chen T., Liu Y., Lv X., Li J., Du G., Ledesma-Amaro R., Liu L. CRISPRi allows optimal temporal control of N-acetylglucosamine bioproduction by a dynamic coordination of glucose and xylose metabolism in Bacillus subtilis. Metab Eng. 2018;49:232–241.

172. Zhang K., Duan X., Wu J. Multigene disruption in undomesticated Bacillus subtilis ATCC 6051a using the CRISPR/Cas9 system. Sci Rep. 2016;6:27943.

173. Zhang K., Su L., Wu J. Enhanced extracellular pullulanase production in Bacillus subtilis using protease-deficient strains and optimal feeding. Appl Microbiol Biotechnol. 2018;102:5089–5103.

174. Gu T., Zhao S., Pi Y., Chen W., Chen C., Liu Q., Li M., Han D., Ji Q. Highly efficient base editing in Staphylococcus aureus using an engineered CRISPR RNA-guided cytidine deaminase. Chem Sci. 2018;9:3248–3253.

175. Chen W., Zhang Y., Yeo W.S., Bae T., Ji Q. Rapid and efficient genome editing in Staphylococcus aureus by using an engineered CRISPR/Cas9 system. J Am Chem Soc. 2017;139:3790–3795.

176. Dong X., Jin Y., Ming D., Li B., Dong H., Wang L., Wang T., Wang D. CRISPR/dCas9-mediated inhibition of gene expression in Staphylococcus aureus. J Microbiol Methods. 2017;139:79–86.

177. Choudhary E., Thakur P., Pareek M., Agarwal N. Gene silencing by CRISPR interference in mycobacteria. Nat Commun. 2015;6:6267.

178. Rock J.M., Hopkins F.F., Chavez A., Diallo M., Chase M.R., Gerrick E.R., Pritchard J.R., Church G.M., Rubin E.J., Sassetti C.M. Programmable transcriptional repression in mycobacteria using an orthogonal CRISPR interference platform. Nat Microbiol. 2017;2:16274.

179. Tan S.Z., Reisch C.R., Prather K.L.J. A robust CRISPR interference gene repression system in Pseudomonas. J Bacteriol. 2018;200

180. Shen J., Zhou J., Chen G.Q., Xiu Z.L. Efficient genome engineering of a virulent Klebsiella bacteriophage using CRISPR-Cas9. J Virol. 2018;92

181. Wang J., Zhao P., Li Y., Xu L., Tian P. Engineering CRISPR interference system in Klebsiella pneumoniae for attenuating lactic acid synthesis. Microb Cell Fact. 2018;17:56.

182. Wang Y.H., Wei K.Y., Smolke C.D. Synthetic biology: advancing the design of diverse genetic systems. Annu Rev Chem Biomol Eng. 2012;4:69–102.
